# The developmental pattern of stimulus and response interference in a color-object Stroop task: an ERP study

**DOI:** 10.1186/1471-2202-9-82

**Published:** 2008-09-05

**Authors:** Ellen MM Jongen, Lisa M Jonkman

**Affiliations:** 1 Department of Cognitive Neuroscience, Section of Biological Developmental Psychology, Maastricht University, Faculty of Psychology, P.O. Box 616, 6200 MD Maastricht, the Netherlands

## Abstract

**Background:**

Several studies have shown that Stroop interference is stronger in children than in adults. However, in a standard Stroop paradigm, stimulus interference and response interference are confounded. The purpose of the present study was to determine whether interference at the stimulus level and the response level are subject to distinct maturational patterns across childhood. Three groups of children (6–7 year-olds, 8–9 year-olds, and 10–12 year-olds) and a group of adults performed a manual Color-Object Stroop designed to disentangle stimulus interference and response interference. This was accomplished by comparing three trial types. In congruent (C) trials there was no interference. In stimulus incongruent (SI) trials there was only stimulus interference. In response incongruent (RI) trials there was stimulus interference and response interference. Stimulus interference and response interference were measured by a comparison of SI with C, and RI with SI trials, respectively. Event-related potentials (ERPs) were measured to study the temporal dynamics of these processes of interference.

**Results:**

There was no behavioral evidence for stimulus interference in any of the groups, but in 6–7 year-old children ERPs in the SI condition in comparison with the C condition showed an occipital P1-reduction (80–140 ms) and a widely distributed amplitude enhancement of a negative component followed by an amplitude reduction of a positive component (400–560 ms). For response interference, all groups showed a comparable reaction time (RT) delay, but children made more errors than adults. ERPs in the RI condition in comparison with the SI condition showed an amplitude reduction of a positive component over lateral parietal (-occipital) sites in 10–12 year-olds and adults (300–540 ms), and a widely distributed amplitude enhancement of a positive component in all age groups (680–960 ms). The size of the enhancement correlated positively with the RT response interference effect.

**Conclusion:**

Although processes of stimulus interference control as measured with the color-object Stroop task seem to reach mature levels relatively early in childhood (6–7 years), development of response interference control appears to continue into late adolescence as 10–12 year-olds were still more susceptible to errors of response interference than adults.

## Background

According to Nigg's [1] taxonomy for inhibitory processing, interference control represents the ability to suppress distracting stimuli, either external or internal, from interfering with current operations of working memory or carrying out a motor response. Interference control is an important component of cognitive control and deficits in interference control are central to several developmental pathologies, for instance attention deficit hyperactivity disorder (for a review, see [2]). Studies on the development of interference control are thus of great importance (for reviews, [3,4]). A common task to study interference control is the Stroop task. In a standard Color-Word Stroop test (for a review, see [5,6]), congruent color words (e.g. the word RED written in red ink) and incongruent color words (e.g. the word RED written in green ink) are presented and participants are asked to identify the color of the ink in which a word is printed while ignoring its identity. Every stimulus thus consists of a relevant stimulus dimension "printed color" that determines the correct response, and a second, irrelevant stimulus dimension "word meaning" that should be ignored. Typically, participants are slower on incongruent trials than on congruent trials, and this reaction time (RT) difference is referred to as the Stroop interference effect. Several researchers have questioned whether interference in the Stroop task occurs at the stimulus level, at the response level, or at both levels. At the stimulus level, the presentation of the irrelevant word might facilitate the encoding or identification of the relevant printed color in congruent trials, or interfere with it in incongruent trials (e.g., [7,8]). At the response level, the presentation of the irrelevant word may automatically activate a response that facilitates response selection in congruent trials but interferes with it in incongruent trials (e.g. [9]). However, these two explanations cannot be discriminated in the standard Stroop interference effect as the measurements of stimulus interference and response interference are confounded when comparing congruent and incongruent trials in a standard color-word Stroop task [5,10]. That is, on congruent trials the irrelevant stimulus dimension (word meaning) is congruent with the relevant stimulus dimension (printed color) and with the response; on incongruent trials the irrelevant stimulus dimension is incongruent both with the relevant stimulus dimension and with the response. The subtraction of the incongruent and the congruent trials thus holds conflict between the two stimulus dimensions as well as conflict at the response level. In the present developmental Stroop study, processes of stimulus interference and response interference will be discriminated to explore the development of stimulus interference control and response interference control in children and adults. Furthermore, event-related potentials (ERPs) will be used to examine the temporal course of these processes and characterize developmental changes in brain activation.

Several studies have utilized the Stroop task to explore the maturational pattern of interference control without disentangling stimulus interference control and response interference control. In a standard color-word Stroop, 7–8 year-olds showed greater Stroop interference than adults [11]. As the classical Stroop task requires proficient reading skills to induce an interference effect, a number of modified Stroop tasks (day-night Stroop, animal Stroop, object Stroop) have been developed to study the development of interference control in childhood (e.g., [12-17]). In a day-night Stroop task that was applied in a group of 3.5–7 year-olds, participants were asked to say "day" to a black card with a white moon, and "night" to a white card with a yellow sun [13]. Interference was largest between 3.5–4.5 years and decreased over age. In an animal Stroop task [17] that was applied in a group of children between 3–16 years old, participants were asked to name the body of animal images (cow, pig, sheep, duck) that could be congruent or incongruent with the presented head. Interference in RT was largest between 3–6 years, decreased over age, and was non-significant between 13–16 years. The largest decrease in RT interference occurred between the group of 5–6 year-olds and the group of 7–8 year-olds. Hanauer and Brooks [14] used a color-word crossmodal (audio-visual) Stroop task in a group of 4–11 year olds and a group of adults and reported an interference effect for RT (but not for accuracy) in each of the age groups (4–5, 6–7, and 9–11 year-olds, and adults) that decreased markedly in size with age. In a follow-up study [15] a picture-word crossmodal (audio-visual) Stroop task was applied in a group of 3–12 year-olds and a group of adults. Now, only the youngest groups (3–5 year-olds and 6–7 year-olds) showed a cross-modal interference effect, whereas the effect was absent in 8–11 year-olds and reversed in adults. Finally, Prevor and Diamond [16] used a color-object Stroop [12] to examine the developmental pattern of interference in a group of 3.5–6.5 year-olds. Line drawings were presented, consisting of familiar objects strongly associated with one particular color (their usual, so-called "canonical color", e.g., heart and red), objects not associated with a particular color (e.g., scissors), and abstract shapes. Line drawings were presented in six possible printed colors, and familiar objects that were associated with a particular color were thus presented either in their canonical color (congruent) or in a different color (incongruent). The task of subjects was to name the printed color of the objects. The results showed clear and equally strong interference (incongruent versus congruent) effects on color naming in RT (but not accuracy rates) in each of the seven age groups (each spanning 6 months). An adjusted manual version of this task was used in the current study. Prevor and Diamond [16] attributed color-object Stroop interference to the prepotent tendency to name and process an object's identity rather than the color in which it is presented. Analogue to the prepotent tendency of word reading in the classical Stroop task, this should be suppressed in order to give the correct response in the color naming task. Prevor and Diamond showed evidence for this tendency as RTs for naming pictured objects were faster than RTs for naming the printed color in which objects were drawn. Furthermore, a recent PET study provided evidence for automatic recognition and processing of objects when the identity of objects was task-irrelevant and correct task performance only required discrimination of global forms (round versus oval) [18]. In addition to this prepotent tendency, color-object interference is suggested to rely on the concurrent activation of characteristic surface features of the object such as its canonical color, when an object's shape and identity are processed [19]. Automatic access to task-irrelevant canonical color knowledge was shown in a detection task when participants were asked to detect a target color or shape [20]. In the Stroop task the activated canonical color is suggested to interfere with the printed color of the object. A Stroop-like delay in RT for naming or manually classifying the printed color of incongruently coloured objects in comparison with congruently coloured objects has been shown in studies with children [12,16] and adults [19,21-23].

The general picture that emerges from the above review of behavioural findings in developmental studies is that despite relevant differences between the Stroop tasks that have been used (e.g. verbal versus manual; auditory versus visual), Stroop interference is stronger in children than in adults, and in the majority of studies shows a decline with age [11,13-15,17]. However, there are large differences in the studied age groups. Furthermore, the age at which interference control seems to be mature appears to be strongly dependent on the type and complexity of the task. In a recent review it was concluded that full maturity of interference control is not reached until roughly 12 years or later [24]. Important in the light of the current study is that in the developmental Stroop studies that were reviewed above, no attempts were made to separate stimulus and response interference. In a number of recent studies with adults, the contribution of both types of interference to the overall behavioural Stroop effect was shown as well as neurobiological independence of these processes [25-29]. De Houwer [25] introduced a two-choice button-press version of the Stroop task to disentangle stimulus and response interference. Congruent and incongruent stimuli were similar to those in a standard Stroop task, but by assigning two colors to each response-button, for example green and red to the left button, and gray and yellow to the right button, three conditions emerged: (1) a congruent condition (C; e.g. the word RED in red ink) in which the irrelevant stimulus dimension (word meaning) was congruent with the relevant stimulus dimension (printed color) and with the response; (2) a stimulus incongruent condition (SI; e.g the word RED in green ink) in which the irrelevant stimulus dimension was incongruent with the relevant stimulus dimension but nevertheless mapped onto the same response as the relevant stimulus dimension and was thus congruent on response level; (3) a response incongruent condition (RI; e.g the word RED in yellow ink) in which the irrelevant stimulus dimension was incongruent both with the relevant stimulus dimension and with the response. It is important to note that only the second condition was new in comparison to a standard Stroop task. Adding this condition allowed for the dissociation of stimulus and response interference by comparing conditions and applying subtractive logic: interference at the stimulus level was measured by a comparison of C and SI trials, and interference at the response level was measured by a comparison of SI and RI. RT results in the study by de Houwer showed evidence in adults for both types of interference, and this has been replicated in other adult studies using the same task [28,29]. In their fMRI study, Van Veen and Carter [29] additionally showed that non-overlapping neural substrates were involved in both types of conflict. The involvement of different brain areas in stimulus and response conflict has also been shown by others using the Stroop task [27] and other types of paradigms [26]. Given the dissociation of effects of stimulus and response interference at the behavioural and neurobiological level in healthy adults, these two types of interference control may follow a different developmental trajectory.

ERP Stroop studies have provided additional measures of interference control. Different from behavioural measures that only provide a snapshot of interference control, ERPs have great temporal sensitivity and provide information about processes of interference control starting directly from stimulus onset. The comparison of incongruent and congruent trials in the classical color-word Stroop task repeatedly has revealed two main modulations [30-39]. First, an enhanced negative component in the incongruent condition as compared to the congruent condition, and a reduced positive component in the incongruent condition as compared to the congruent condition have been reported between 350–500 ms after stimulus-onset over frontal, fronto-central, central, and parietal areas. In a difference wave of the incongruent condition minus the congruent condition these modulations both result in a negative amplitude difference, and that has been referred to as the N450 or N500. Second, following this negative amplitude difference, an amplitude enhancement of a late positive component in the incongruent condition as compared to the congruent condition has been reported, that has been suggested to arise from a distributed network involving lateral frontal, parietal, and occipital sites [34]. Although both amplitude modulations are thought to be involved in conflict processing, and have been shown sensitive to the degree of conflict [30,36,38], the exact functional interpretation of these amplitude modulations has not been elucidated. Some suggestions have been made, relating the negative amplitude difference to conflict detection [31,32,34,35,38,39] and the need to suppress irrelevant conflicting information [36]. Furthermore, the late amplitude enhancement has been related to conflict resolution and the processing of relevant information that is used to guide response selection in incongruent trials [31,34,36], and to processes of response selection [39]. Importantly, in one of these studies the distinction between stimulus and response conflict was examined in a counting Stroop task by manipulating conflict at the combined stimulus-and-response level and conflict solely at the stimulus level [38]. The two modulations described before (negative amplitude difference and enhanced positive component), that are normally evoked by incongruent (versus congruent) stimuli were elicited in both instances which suggests that these component modulations are a reflection of both stimulus and response interference. In a series of recent numerical Stroop ERP studies, [40-42] an ERP based index of motor processing, the lateralized readiness potential (LRP), was used to investigate stimulus and response interference. In these studies processes at the stimulus level were defined as those that occurred before the LRP, and processes at the response level were defined as those that occurred after LRP onset. Evidence was shown for interference at the stimulus level and interference at the response level. In one of these studies the developmental pattern of stimulus and response interference was examined in 9 and 11 year-old children and adults [42]. Based on ERP measures it was concluded that interference in children in comparison with adults was more due to response processes than to stimulus processes and that this difference was probably due to trouble with inhibition of response tendencies in children.

**Figure 1:**
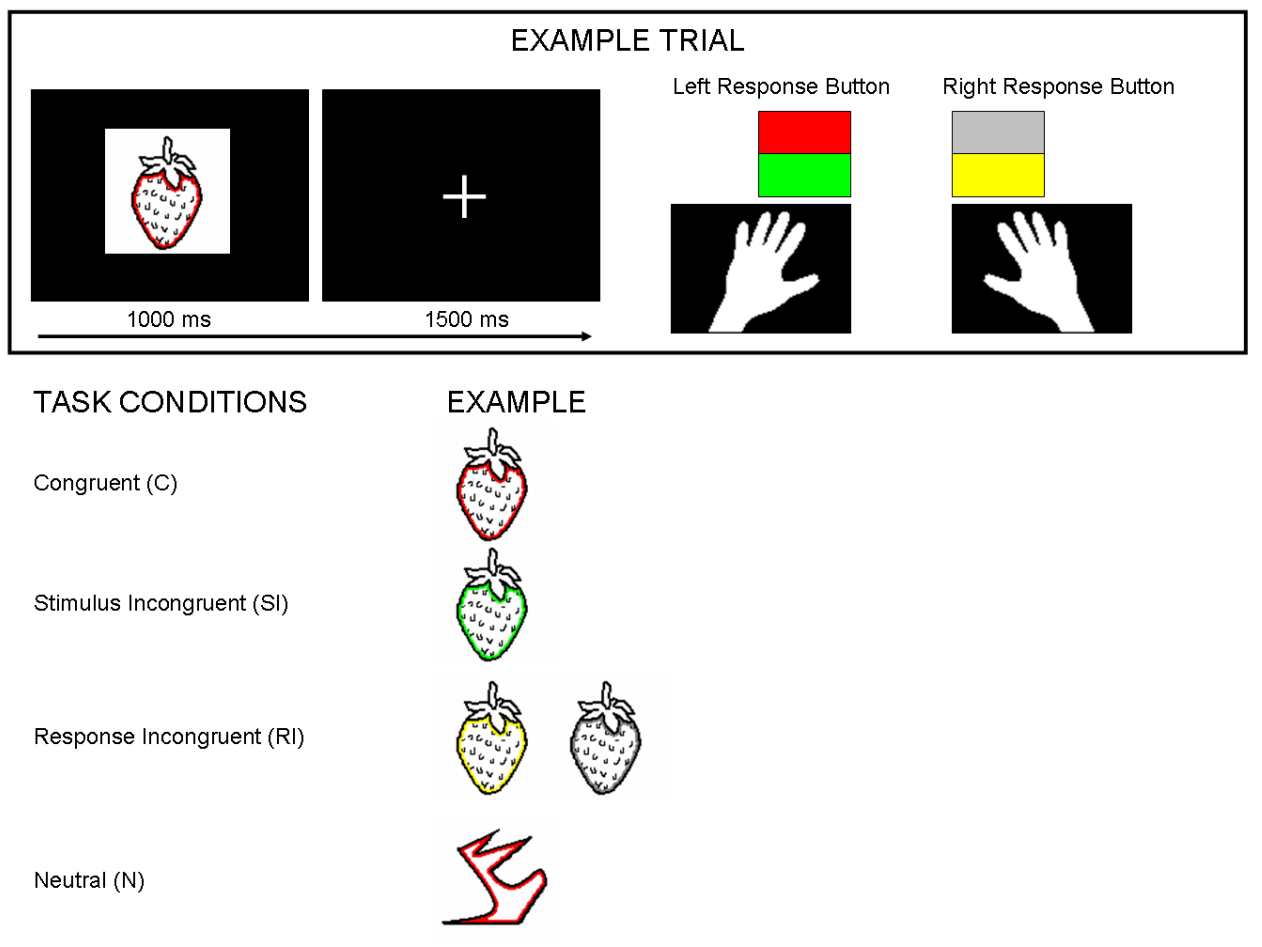
**Trial structure (A) and task conditions (B)**. (A) Schematic illustration of a trial. Stimuli are not to scale. Subjects were instructed to discriminate the outline of every object and respond to it as fast and accurately as possible by pressing the correct response button. Response buttons are coloured here for demonstration purposes; in reality they were white coloured and participants learned to associate each button with two colors in the practice session. (B) Schematic example of the four conditions. Incongruent stimuli are defined as either stimulus incongruent (SI) or response incongruent (RI) depending on both the canonical color of a stimulus (in this example: red) and the mapping of the colors to the response buttons (in this example: red and green to the left button, gray and yellow to the right button). In the SI condition, the incongruent color was mapped onto the same response button as the object's canonical color. In the RI condition, the object was presented in one of the incongruent colors that were mapped onto the response button opposite to the button associated with the object's canonical color. In the neutral condition abstract shapes were presented in one of the four colors.

The aim of the present study was to determine whether interference at the stimulus (perceptual) level and interference at the response (selection) level are subject to distinct maturational patterns across childhood. Therefore, children (aged 6–12 years) and adults performed a manual Color-Object Stroop task [16] that was designed to separate stimulus and response interference using the procedure applied by de Houwer that was described before [25] (see Figure 1). Similar to Prevor and Diamond, line drawings of familiar objects with a canonical color (e.g., a strawberry) were presented either in their canonical color (congruent: e.g., red strawberry) or in a different color (incongruent: e.g., blue strawberry). In addition, abstract shapes were presented in the neutral condition. The task of subjects was to classify the printed color of the stimuli by pressing one of two buttons. In the object-Stroop task the irrelevant stimulus dimension "canonical color" of the object interferes with the relevant stimulus dimension "printed color" of the object. By associating two colors to each response button a congruent (C), stimulus incongruent (SI), and response incongruent (RI) condition emerged. In the SI condition the irrelevant stimulus dimension (canonical color) is incongruent with the relevant stimulus dimension (printed color), but both stimulus dimensions ask for the same response. Consequently, there is no conflict at response level. In the RI condition the irrelevant stimulus dimension (canonical color) again is incongruent with the relevant stimulus dimension (printed color) but in addition the stimulus dimensions activate conflicting responses based on learned stimulus-response associations. Given that stimulus incongruent and congruent stimuli are both congruent at the response level, interference at the stimulus level can be measured by a comparison of SI-C. Given that stimulus incongruent stimuli and response incongruent stimuli are both incongruent at the stimulus level and only response incongruent stimuli are incongruent at the response level, interference at the response level can be measured by a comparison of RI-SI. High-density 60-channel ERPs provided additional temporally sensitive measures of stimulus interference and response interference immediately after stimulus onset. In the study by Prevor and Diamond only children between 3.5–6.5 years were tested, but previous studies have shown that maturational differences in response inhibition and conflict control still occur between 6–7 and 10–12 years of age [43,44]. The present study extends the study by Prevor and Diamond by testing children between 6–12 years as well as a group of adults. Small age ranges (6–7, 8–9, and 10–12 year-old) were used, permitting a detailed investigation of the trajectory of cognitive developmental changes. Finally, to our knowledge, this is the first ERP study that uses a color-object Stroop rather than the standard color-word Stroop task. In an fMRI study that compared the performance on the color-word and color-object Stroop task, patterns of neural activation in both tasks partly overlapped, but differences were also shown [21]. Whereas activation of prefrontal areas, suggested to be related to the selection of task-relevant color information, was similar in both tasks, there were differences in the posterior pattern of activation, suggested to be related to the selection of irrelevant information (words versus objects). More specifically, the color-object task activated occipito-temporal areas that were not shown active in the color-word Stroop.

## Methods

### Participants

Twenty-one adults (age 18.6–28.8, mean age 21.7, 11 female) and fifty-seven children (age 6.4–12.8, mean age 9.1, 29 female) participated in the study. Three children were excluded from the analyses because of technical problems. All adults were students from Maastricht University and were paid for participation. Children were allocated to one of three age groups: 18 children participated in the 6–7 group (age 6.4–7.8, mean age 7.0, 8 female); 19 children participated in the 8–9 group (age 8.0–9.8, mean age 9.0, 9 female), and 17 children participated in the 10–12 group (age 10.1–12.8, mean age 11.2, 10 female). Children were recruited from two elementary schools and received a present for their participation in the experiment. The experimental methods had ethical approval from the institutional ethics committee. Informed consent was obtained from all adult subjects and the parents of the children.

An estimation of full-scale IQ was derived from the individual scores on two subtests (vocabulary and block design) of the Dutch version of the Wechsler Adult Intelligence Scale (WAIS-III) and of the the Wechsler Intelligence Scale for Children (WISC-III). The mean reliability and validity of this IQ-score when compared to the complete test is .9 for both scales [45,46]. The mean IQ-score was 112.6 (range 91–132) for the 6–7 group, 105.9 (range 88–132) for the 8–9 group, 100.7 (range 80–123) for the 10–12 group, and 117.0 (range 100–143) for adults. The difference between groups was significant F(3, 71) = 7.8, p < .0005. Post-hoc tests showed a significant difference between the 6–7 group and the 10–12 group (t(33) = 3.2, p = .003), and a difference between the group of adults on the one hand and the 8–9 group (t(38) = 3.1, p = .004) and 10–12 group (t(36) = 4.5, p < .0005) on the other hand.

To measure the presence of any attention and hyperactivity/impulsivity problems, adults filled out the Self-Report form of the ACTeRS [47]. This form consists of 35 items; 10 items to assess problems of Attention, 10 items to assess problems of Social Adjustment (the latter not used in present study), and 15 items to assess problems of Hyperactivity/Impulsivity. The raw scores were converted to gender-neutral percentile ranks and t-scores. A lower score on the ACTeRS is associated with enhanced problem behavior. The ACTeRS was standardized based on a total of 1012 cases; a t-score of 46 or higher on the Attention and Hyperactivity/Impulsivity subscales indicates a score within the 70% range of the population scores. Subjects diagnosed with ADHD scored in the lowest 10% of the population range, corresponding to a t-score below 41 on both scales. All adults were included as scores were never within the lowest percentile range. The mean normalized T-score was 52 (range 43–60) for the Attention subscale, and 50 (range 43–63) for the Hyperactivity/Impulsivity subscale.

To measure the presence of any attentional problems, internalizing behavioral disorders, or externalizing behavioral disorders in the children, parents filled out the Child Behavior Check List (CBCL; [48]). The clinical range is reflected by a t-score of 70 or higher for the Attention subscale, and a t-score of 63 or higher for the Internalizing and Externaling subscales. The borderline clinical range is reflected by a t-score between 65–69 for the Attention subscale, and a t-score between 60–63 for the Internalizing and Externaling subscales. All children were included as scores of the subscales were never within the clinical range.^1^

### Stimuli

Stimuli were line drawings of sixteen familiar objects^2 ^that were each strongly associated with one color, their canonical color (e.g., strawberry and red), and four abstract shapes. Four colors were used; red, green, yellow, or gray. Line drawings were drawn in black, outlined in one of the colors, and presented in a white square (4.5 cm × 4.5 cm) on a black background. The fixation cross was presented in white. There were an equal number of familiar objects for each of the four canonical colors; four objects for each color. By presenting each of the objects and abstract shapes in the task in each of the four colors, there were 80 "unique" stimuli and these were presented repeatedly, as explained below in the task description.

### Task description

The task is illustrated in Figure 1. The Stroop task was partly similar to the one used by Prevor and Diamond [16] that was explained in the introduction. Two response buttons, a left and a right one, were used, and two colors were assigned to each button. As shown in Figure 1A, on every trial, a line drawing was presented for 1000 ms, followed by an inter-stimulus interval (ISI) during which a fixation cross was presented for 1500 ms. Participants were instructed to discriminate the outline color of an object by pressing the correct response button. They were asked to respond fast and accurately while maintaining central eye fixation.There were four task conditions, and these are illustrated in Figure 1B. In the neutral condition (N), abstract shapes were presented in one of the colors. In the congruent condition (C), familiar objects were outlined in their canonical color. In the stimulus incongruent condition (SI), a familiar object was presented in the incongruent color that was mapped onto the same response button as the object's canonical color. In the response incongruent condition (RI), a familiar object was presented in one of the incongruent colors that were mapped onto the response button opposite to the button associated with the object's canonical color.

Task instructions always were presented visually (on the computer screen) as well as verbally. The experiment consisted of a practice session followed by 320 experimental trials. Experimental trials were presented in five 64-trial experimental blocks. In each of these blocks the four conditions (N, C, SI, RI) were equiprobable (16 trials for each condition) and were presented randomly. The practice session consisted of four phases. Every practice phase was repeated until a performance criterion of 75% correct was reached. During the first phase, "the object identification phase", participants were asked to name aloud every object to ensure that they were familiar with all objects. During the second phase, "the color identification phase", colored rectangles of the four colors that were used in the experiment were presented on a black background and participants were asked to name aloud the color name to ensure that subjects were familiar with all colors. During the third phase, the four colors used in the experimental session were associated with the response buttons; two colors were associated with the left button and two colors were associated with the right button. This was done by assigning each of the colors to a response button and by asking participants to respond fast and accurately to colored rectangles presented at the centre of the screen (1000 ms) by pressing the correct button. Forty trials were presented. During the fourth and final practice phase, one block of 64 trials, similar to an experimental block, served to practice the main task. During the third and the fourth practice phase, computerized feedback was given on every trial consisting of a short text message (correct, false, or faster). No feedback was given in the experimental task.

### Procedure

Testing was done at the elementary school (children) or university (adults). At arrival, adults were asked to fill out the ACTeRS questionnaire. After the preparations for the EEG recordings, participants performed on a blink calibration task. After this calibration task the Stroop task^3 ^was presented. After removal of the EEG cap, the vocabulary and block design subtests of the WAIS-III (adults) or WISC-III (children) were performed. Tasks were presented on a VGA monitor that was placed at a viewing distance of 50 cm. ERTSVIPL V3.37b [49] controlled the tasks.

### EEG recording and ERP analyses

Electroencephalographic (EEG) activity (bandpass 0.05–120 Hz), digitized at 500 Hz, was recorded continuously via Brainvision Analyzer from 60 scalp locations (Fp1, Fpz, Fp2, AF7, AF3, AF4, AF8, F7, F5, F3, F1, Fz, F2, F4, F6, F8, FT7, FC5, FC3, FC1, FCz, FC2, FC4, FC6, FT8, T7, C5, C3, C1, Cz, C2, C4, C6, T8, TP7, CP5, CP3, CP1, CPz, CP2, CP4, CP6, TP8, P7, P5, P3, P1, Pz, P2, P4, P6, P8, PO7, PO3, PO4, PO8, O1, Oz, O2, and right mastoid A2) using tin electrodes mounted on an elastic cap (Quik-Cap). Horizontal and vertical eye movements were recorded from tin electrodes placed at outer canthi of both eyes, and above and below the left eye, respectively. Electrode impedance was kept below 10 kΩ. AFz was used as the ground. During recording the left mastoid (A1) was used as a reference; for data-analysis electrodes were re-referenced to the average of right and left mastoids.

ERP analysis was done in Neuroscan 4.3. To prevent rejection of too many trials, instead of rejecting trials that contained eyeblinks from the analyses, blink activity was subtracted from the EEG signal by applying a regression procedure incorporated in Neuroscan software [50]. A blink calibration task was used to evoke eye-blinks that were not linked to the experimental task. In the calibration task, spontaneous blinks were promoted by demanding constant fixation to detect slow color changes of a fixation cross. Offline, blinks were manually detected (a minimum of 20 blinks served as a criterion) for every subject and used to determine the average blink response for every subject. In the regression procedure, by relating blink activity at the VEOG channel with EEG activity at the different EEG channels the transfer of blink activty at every separate EEG channel was determined and expressed in regression coefficients for every electrode. After carefully checking the standard deviations (across 20 trials) and topography of these coefficients (strong frontal fields), these coefficients were used to remove eye-blink activity from the EEG. Data were re-filtered with a low pass filter of 30 Hz (48 dB/oct.). Epochs were made -200 ms to 1000 ms relative to stimulus onset. Incorrect response trials and trials with artifacts in the EEG signal exceeding a voltage of +/-125 μV were excluded from the analyses. ERPs were computed relative to the 200 ms baseline for each subject, for each of the four conditions (N, C, SI, RI). Grand averages were then computed for each of the groups, for each of the four conditions. The N condition was later excluded from the analyses as it appeared not to be a good comparison condition because of the abstract shapes (see Figure 1) and the deviating response patterns elicited by them in especially young children (i.e., delayed response times and enhanced errors).

The minimum number accepted trials in every condition (max. 80) was 30, based on Thomas et al. [51] where it was shown that when the number of trials included in the average was lower than 28, peak amplitude analyses were most strongly biased (but note that in the present study only mean area amplitude analyses over larger time-windows were done, and these are less sensitive to such biases and trial differences between conditions). After exclusion of trials with a voltage exceeding +/-125 μV or errors, in the group of adults, 6–7 year-old, 8–9 year-old, and 10–12 year-old children, respectively, an average (range, S.D.) of 78.5 (74–80, 1.8), 62.0 (45–72, 8.0), 68.1 (51–78, 8.1), 73.1 (48–79, 7.2) trials in the C condition; 78.0 (72–80, 2.3), 63.6 (44–76, 8.5), 67.6 (47–79, 8.5), 73.8 (46–80, 7.9) trials in the SI condition, and 77.5 (73–80, 1.8), 61.1 (33–74, 9.9), 63.3 (51–78, 7.9), 68.2 (43–78, 8.6) trials in the RI condition remained for analyses.

### Statistical analyses

#### Behavioral data

A logarithmic transformation was applied to RTs prior to all analyses to reduce the effect of baseline differences between age groups [52]. The square roots of error percentages were analyzed separately for omission errors (misses) and commission errors (pressing the wrong response button). As explained in the introduction, interference at the stimulus level was analyzed by comparing effects in the SI condition and the C condition, and interference at the response level was analyzed by comparing effects in the SI condition and the RI condition. Mean log-transformed reaction time data^4 ^and the square roots of error percentages were analyzed using an overall 4 (Group) × 3 (Condition: C, SI, RI) ANOVA. In case of a main effect of Condition or an interaction of Group × Condition, two planned ANOVAs were performed to investigate the developmental pattern of stimulus interference (4 (Group) × 2 (Condition: C, SI)), and response interference (4 (Group) × 2 (Condition: SI, RI)). In case of a significant Group × Condition interaction, Bonferroni-corrected post-hoc between-group comparisons were carried out to further examine group differences in interference. In addition, paired-samples t-tests were carried out to test for interference effects within every group. IQ-score was entered in all these analyses as a continuous predictor variable.

#### ERPs

For the ERP analyses, based on research questions mentioned in the introduction, specific planned analyses were performed to investigate Group (age) differences in ERP responses to stimulus interference (SI versus C) and response interference (RI versus SI).

**Figure 2:**
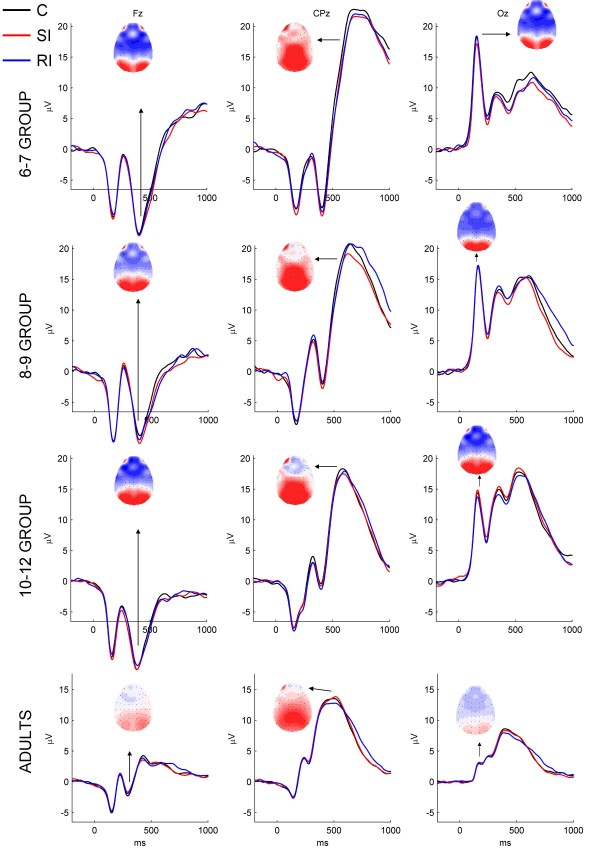
**Grand-averaged ERPs for all conditions**. Grand-averaged ERPs for congruent (C; black line), stimulus incongruent (SI; red line), and response incongruent (RI; blue line) conditions at midline electrodes Fz, CPz, and Pz in each of the four age groups. Topographical maps are for the C condition and show similar scalp distributions for the N4 (shown at Fz), P3 (shown at CPz), and P1 (shown at Oz) component in each of the age groups. Scalp distributions across groups were also similar for the SI condition and the RI condition, but these were left out for reasons of space and redundancy.

The time windows and Electrodes of interest to be included in the analyses were determined following a number of steps. Because of a lack of developmental ERP studies using a similar color-object Stroop task, the choice of time windows and electrodes was mainly based on the acquired data and inspection of Grand Average waves as well as SI-C and RI-SI difference waves. First, Grand Average ERPs in the different groups across midline electrodes were inspected (see Figure 2). Similar to other Stroop studies, the ERPs of children showed a clear negative component distributed over frontal-central and parietal electrodes, around 400–560 ms that resembles an "N400" component described in the Stroop literature (see Figure 2, Fz, for topographical maps). This negative component was followed by a broad positive component with a central-parietal-occipital distribution starting around 500 ms and ending around 900–1000 ms (see Figure 2, CPz, for topographical maps). At the occipital electrodes, a clear P1 response was present around 160–170 ms in all children groups (see Figure 2, Oz, for topographical maps). In adults, the same activity was present with similar topographical distributions, but the activity was smaller and with earlier latencies; the negative component had its maximum around 300 ms and the centro-parietal positive component occurred in a window from 400–800 ms. The P1 was smaller in amplitude but occurred only about 10 ms earlier in adults than children.

**Figure 3:**
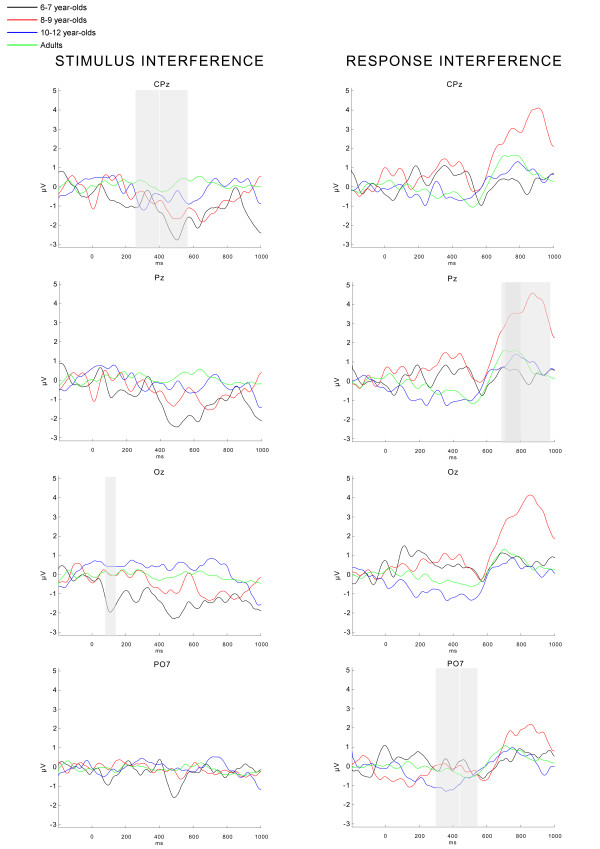
**Difference waves for stimulus interference and response interference**. Difference waves for stimulus interference (SI – C) and response interference (RI-SI) for each of the four age groups at midline electrodes CPz, Pz, Oz, and electrode PO7. These waves were computed to determine the latency windows of stimulus interference and response interference. Gray-colored bars indicate the two effects (described in the text) for stimulus interference and response interference. For stimulus interference the effect overlapping the negative component (N4) and the positive component (P3) is shown at CPz. The first and the second gray bar indicate the latency window used for adults (260–400 ms) and children (400–560 ms), respectively. The P1 effect for stimulus interference is shown at Oz at a similar latency window for children and adults (80–140 ms). For response interference the effect at the P3-like component lateralized over parietal sites is shown at PO7. The second gray bar indicates the latency window (440–540 ms) used for all the groups; the first gray bar indicates the earlier latency window (300–440 ms) during which the effect already started in 10–12 year-olds. The late response interference effect at the descending flank of the P3 is indicated at Pz. The first and the second gray bar overlap and indicate the latency window used for adults (680–800 ms) and children (700–960 ms), respectively. The effects were not limited to the electrodes shown here; see the text for the exact selection of electrodes used in statistical tests.

The second step was to determine the latency windows in which differences in stimulus (SI-C) interference or response interference (RI-SI) effects were present within groups; latency differences are known to occur due to development. Therefore, difference waves were computed at midline electrodes (see Figure 3; note that because of scale inflation these difference waves look noisier than the grand average waves). Inspection of these difference waves led to the detection of four effects of stimulus interference (SI-C) and response interference (RI-SI) that were further tested: 1) an effect of stimulus interference on the P1, occurring at similar latencies in all groups (see Figure 3, Oz). Therefore, a window from 80–140 ms was adopted; 2) an effect of stimulus interference overlapping the negative component (N4) and the positive component (P3) (see Figure 3, CPz). This effect occurred in children in a window of 400–560 ms, and due to a latency shift of the N4 and P3 in a window from 260–400 ms in adults; 3) an effect of response interference on the positive (P3-like) component around 440–540 ms (see Figure 3, PO7), and 4) a late effect of response interference on the descending flank of the positive component that occurred in a time window from 680–800 ms in adults, and from 700–960 ms in children.

**Figure 4:**
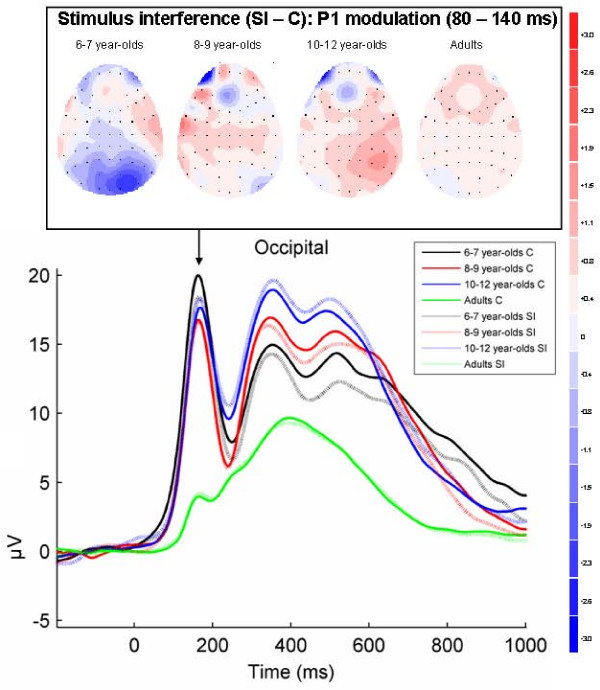
**Stimulus interference effects on occipital P1 amplitude (80–140 ms)**. Grand-averaged ERPs for congruent (C) and stimulus incongruent (SI) conditions at an occipital average of electrodes (Oz, O2, PO8) in each of the four age groups. The arrow indicates the P1 component. Topographical maps of the voltage difference for the SI minus C condition (blue: negative difference; red: positive difference) indicating stimulus interference show a negative amplitude difference (80–140 ms) at central and right-hemispheric occipital sites reflecting an amplitude reduction of the P1 component that was most pronounced in 6–7 year-olds.

**Figure 5:**
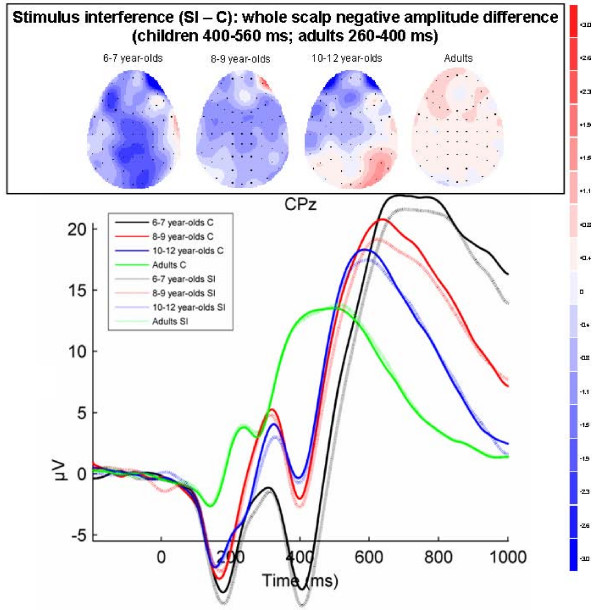
**Stimulus interference effects overlapping the negative (N4) and positive (P3) components (adults 260 – 400 ms; children 400 – 560 ms)**. Grand-averaged ERPs for congruent (C) and stimulus incongruent (SI) conditions at CPz in each of the four age groups. Topographical maps of the voltage difference for the SI minus C condition (blue: negative difference; red: positive difference) indicating stimulus interference show a negative amplitude difference (adults: 260–400 ms; children: 400–560 ms) reflecting the amplitude enhancement of the N4 component and the amplitude reduction of the P3-like component widely distributed over the scalp and most pronounced in 6–7 year-olds that decreases with age.

**Figure 6:**
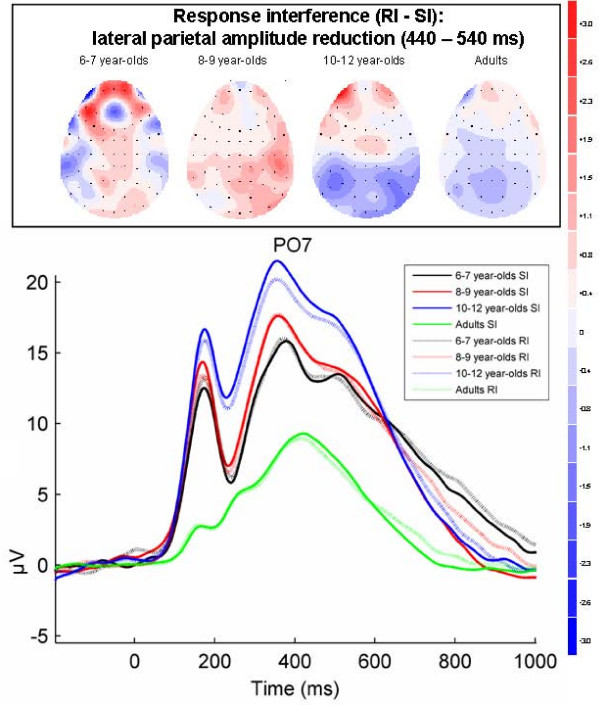
**Response interference effect on parietal-occipital positive component (440–540 ms)**. Grand-averaged ERPs for stimulus incongruent (SI) and response incongruent (RI) conditions at PO7 in each of the four age groups. Topographical maps of the voltage difference for the RI minus SI condition (blue: negative difference; red: positive difference) indicating response interference show a negative amplitude difference (440–540 ms) reflecting the amplitude reduction of the P3-like component over lateral parietal sites in 10–12 year-olds and adults.

**Figure 7:**
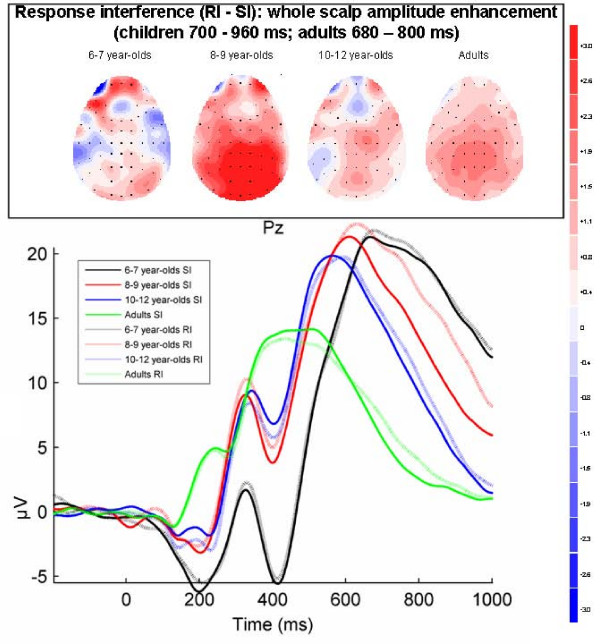
**Response interference effects on positive component across whole scalp (adults 680–800 ms; children 700–960 ms)**. Grand-averaged ERPs for stimulus incongruent (SI) and response incongruent (RI) conditions at Pz in each of the four age groups. Topographical maps of the voltage difference for the RI minus SI condition (blue: negative difference; red: positive difference) indicating response interference show a positive amplitude difference (adults: 680–800 ms, children: 700–960 ms) reflecting the amplitude enhancement of the descending flank of the positive P3-like component widely distributed over the scalp in all groups.

In the third step Electrodes to be included in the analyses of these four different stimulus interference and response interference effects were determined. For this purpose, topographic maps were made of the difference activity in the above mentioned time windows in all groups. For the stimulus interference effect overlapping the negative component (N4) and the positive component (P3), and for the response interference effect on the late descending flank of the positive component a broad scalp distribution across medial electrodes was visible in the topographic maps (see Figures 5 and 7). Therefore, in these analyses 18 electrodes were included (Fz-F1-F2, FCz-FC1-FC2, Cz-C1-C2, CPz-CP1-CP2, Pz-P1-P2, Oz-O1-O2). The stimulus interference P1 effect (in 6–7 year-olds) was clearly lateralized at the right occipital hemisphere (see Figure 4), and therefore electrodes Oz, O2, and PO8 were included in this analysis. The response interference effect around the maximum of the positive component that was most pronounced in 10–12 year-olds and adults had a lateralized distribution across parietal-occipital electrodes (see Figure 6) and therefore 8 bilateral parietal electrodes (P1, P3, P5, P7, and P2, P4, P6, P8) and 2 bilateral parietal-occipital (PO7, PO8) electrodes were included in this analysis.

Mean voltage values in the specified time windows from the subjects in the different groups were entered into a mixed design analysis of variance (ANOVA). In each of these analyses Group (4: 6–7, 8–9, 10–12 year-olds, and adults) was included as between-subjects factor. Condition (2: CO, SI for stimulus interference; SI, RI for response interference) and Electrodes were included as within-subjects factors. In the analysis of the response interference effect on the P3-like component in the 440–540 ms window an extra within-subjects factor Hemisphere (2: left, right) was included because of lateralized distributions. Significant interactions involving the factors Group × Condition × Electrode (or Hemisphere for the response interference effect on the positive component) were followed by tests for Group × Condition effects at the separate electrodes (or groups of electrodes; frontal, central, centro-parietal, etc.). In case of no significant interactions of the Group × Condition effects with Electrodes, further analyses were performed including all electrodes. All Group × Condition interactions were followed by tests of interference effects in the separate groups. For all analyses, P-value was set at 0.05, corrected for deviations from sphericity (Greenhouse-Geisser epsilon correction). The corrected F- and probability values, the uncorrected degrees of freedom, and the Greenhouse-Geisser epsilon are reported.

## Results

### Behavioral performance

There was no interaction of Group and IQ score in any of the error or RT analyses. Therefore, analyses were run without the interaction component.

Averages of untransformed error percentages and RT data for the different groups and conditions are presented in Table 1 and Table 2, respectively.

**Table 1:** Mean Percentage of Commission Errors (PC) and Omission Errors (PO) for every group as a function of Trial Type

Trial type	6–7 year-olds				8–9 year-olds				10–12 year-olds				Adults			
	PC		PO		PC		PO		PC		PO		PC		PO	
	M	SD	M	SD	M	SD	M	SD	M	SD	M	SD	M	SD	M	SD
Neu	15.63	7.33	2.15	3.95	12.30	7.34	1.38	2.35	8.46	6.30	0	0	2.08	2.72	.06	.27
C	13.06	6.28	2.15	3.37	9.14	6.28	0.66	1.21	4.26	2.38	0.15	0.61	1.61	1.90	.06	.27
SI	9.79	5.91	2.78	3.65	9.08	6.70	1.45	2.22	4.04	2.95	0.22	0.66	2.20	2.47	0	0
RI	14.86	8.93	2.78	5.21	14.87	7.81	1.05	1.83	9.71	6.12	0.59	1.09	2.92	2.18	0	0
SI-C	-3.26	6.35	.63	1.62	-.07	3.60	.79	2.36	-.22	2.97	.07	.93	.60	1.96	-.06	.27
RI-SI	5.07	7.91	0	2.19	5.79	4.66	-.39	2.13	5.66	5.57	.37	1.23	.71	1.61	0	0

**Table 2:** Mean Reaction Time (RT) for every group as a function of Trial Type

Trial type	6–7 year-olds		8–9 year-olds		10–12 year-olds		Adults	
	M	SD	M	SD	M	SD	M	SD
Neu	931.9	151.5	804.4	155.6	673.0	99.9	530.7	53.6
C	896.7	130.0	776.4	130.0	660.2	92.4	529.9	48.7
SI	901.5	159.8	789.8	127.1	658.5	90.2	530.3	45.4
RI	930.7	147.4	824.1	137.0	686.9	101.1	554.8	58.1
SI-C	4.7	55.5	13.4	57.9	-1.7	25.5	0.40	13.7
RI-SI	29.2	49.5	34.3	47.8	28.4	34.8	24.6	28.5

#### Omission errors

As shown in Table 1, the average percentage of *omission *errors was very low, and the overall ANOVA including C, SI, and RI stimuli only showed an effect of Group (F(3, 70) = 9.0, p < .0005, *η*_*p*_^2 ^= .28), indicating an overall linear decrease of misses with age. The latter was confirmed by a significant linear contrast (F(1, 70) = 24.7, p < .0005, *η*_*p*_^2 ^= .26) in the absence of a quadratic contrast (F(1, 70) = 2.4, p = .13, *η*_*p*_^2 ^= .03) or cubic contrast (F(1, 70) < 1, p = .55, *η*_*p*_^2 ^= .005). Since there was no effect of Condition (F(2, 140) = 2.1, p = .13, epsilon = .996, *η*_*p*_^2 ^= .03) or Group × Condition (F(6, 140) = 1.6, p = .14 *η*_*p*_^2 ^= .07), no further planned analyses were carried out for stimulus interference and response interference.

#### Commission errors

The overall ANOVA for commission error data including C, SI, and RI stimuli, showed a main effect of Condition (F(2, 140) = 36.8, p < .0005, epsilon = .97, *η*_*p*_^2 ^= .35), and Group (F(3, 70) = 27.5, p < .0005, *η*_*p*_^2 ^= .54), as well as an interaction of Group × Condition (F(6, 140) = 3.2, p = .01, *η*_*p*_^2 ^= .12). As announced in the introduction, further planned contrasts were carried out to test for group differences in stimulus interference (C versus SI) and response interference (SI versus RI).

The planned analysis of *stimulus interference *showed a main effect of Group (F(3, 70) = 25.8, p < .0005, *η*_*p*_^2 ^= .53), indicating a linear decrease in commission error percentages with age, as confirmed by a significant linear contrast (F(1, 70) = 76.0, p < .0005, *η*_*p*_^2 ^= .52) in the absence of a quadratic contrast (F(1, 70) < 1, p = .69, *η*_*p*_^2 ^= .002) or cubic contrast (F(1, 70) < 1, p = .33, *η*_*p*_^2 ^= .01). Although there was no effect of Condition (F(1, 70) = 2.1, p = .16, epsilon = 1.0, *η*_*p*_^2 ^= .03), there was an interaction of Group × Condition (F(3, 70) = 3.8, p = .01, *η*_*p*_^2 ^= .14). Further group comparisons showed a between-group difference in stimulus interference only between 6–7 year-olds and adults (p = .015, *d *= .96), and not between any of the other groups (.29 <p's < = 1.0). Follow-up within-group t-tests showed a condition effect only in the 6–7 group (t(17) = 2.5, p = .02, *d *= .59), but contrary to prediction more commission errors were made in the C condition than in the SI condition. No stimulus interference effects on commission errors were found in the other groups (8–9 group, t(18) < 1, p = .86, *d *= .04; 10–12 group, t(16) < 1, p < .40, *d *= .21, adults (t(20) = 1.6, p = .14, *d *= .34).

The planned analysis of *response interference *similarly showed a main effect of Group (F(3, 70) = 20.7, p < .0005, *η*_*p*_^2 ^= .47) indicating a linear decrease in commission error percentages with age, as confirmed by a significant linear contrast (F(1, 70) = 58.3, p < .0005, *η*_*p*_^2 ^= .45) in the absence of a quadratic contrast (F(1, 70) = 2.3, p = .14, *η*_*p*_^2 ^= .03) or cubic contrast (F(1, 70) < 1, p = .33, *η*_*p*_^2 ^= .01). In addition there was a main effect of Condition (F(1, 70) = 54.6, p < .0005, epsilon = 1.0, *η*_*p*_^2 ^= .44) and an interaction of Group × Condition (F(3, 70) = 3.0, p = .04, *η*_*p*_^2 ^= .12). Further group comparisons showed a between-group difference in response interference only between 10–12 year-olds and adults (p = .04, *d *= .92), and not between any of the other groups (.32 <ps < = 1.0). Follow-up within-group t-tests showed a higher number of errors for the RI in comparison with the SI condition in all children (6–7 group, t(17) = 3.2, p = .01, *d *= .75; 8–9 group, t(18) = 4.8, p < .0005, *d *= 1.1; 10–12 group, t(16) = 4.4, p < .0005, *d *= 1.1), but in adults this effect only approached significance (t(20) = 1.9, p = .07, *d *= .41).

#### Reaction time

The overall ANOVA (including C, SI, and RI) for reaction time data showed a main effect of Group (F(3, 70) = 54.2, p < .0005, *η*_*p*_^2 ^= .70) indicating a linear decrease in RT with age, as confirmed by a significant linear contrast (F(1, 70) = 161.5, p < .0005, *η*_*p*_^2 ^= .70) in the absence of a quadratic contrast (F(1, 70) < 1, p = .43, *η*_*p*_^2 ^= .01) or cubic contrast (F(1, 70) < 1, p = .82, *η*_*p*_^2 ^= .001). In addition, there was a main effect of condition (F(2, 140) = 36.9, p < .0005, epsilon = .99, *η*_*p*_^2 ^= .35), indicating an increase in RT from C (715.8 ms) to SI (720.0 ms) to RI (749.1 ms). There was no interaction between group and condition (F(6, 140) < 1, p = .89, *η*_*p*_^2 ^= .02).

The planned analysis of the development of *stimulus interference *(C versus SI) showed a main effect of Group (F(3, 70) = 54.8, p < .0005, *η*_*p*_^2 ^= .70) indicating a linear decrease in RT with age, as confirmed by a significant linear contrast (F(1, 70) = 163.5, p < .0005, *η*_*p*_^2 ^= .70) in the absence of a quadratic contrast (F(1, 70) < 1, p = .44, *η*_*p*_^2 ^= .01) or cubic contrast (F(1, 70) < 1, p = .90, *η*_*p*_^2 ^< .0005). There was no effect of Condition (F(1, 70) < 1, p = .32, epsilon = 1.0, *η*_*p*_^2 ^= .01), and no interaction of Group × Condition (F(3, 70) < 1, p = .75, *η*_*p*_^2 ^= .02).

The planned analysis of the development of *response interference *similarly showed a main effect of Group (F(3, 70) = 52.9, p < .0005, *η*_*p*_^2 ^= .69) indicating a linear decrease in RT with age, as confirmed by a significant linear contrast (F(1, 70) = 157.4, p < .0005, *η*_*p*_^2 ^= .69) in the absence of a quadratic contrast (F(1, 70) < 1, p = .41, *η*_*p*_^2 ^= .01) or cubic contrast (F(1, 70) < 1, p = .73, *η*_*p*_^2 ^= .002). Reaction times for the RI condition were significantly slower than RTs for the SI condition, as shown by a main effect of Condition (F(1, 70) = 45.2, p < .0005, epsilon = 1.0, *η*_*p*_^2 ^= .39). The absence of an interaction of Group × Condition (F(3, 70) < 1, p = .94, *η*_*p*_^2 ^= .006) shows that response interference effects on RT were equally strong in all age groups.

In sum, there were no stimulus interference effects on RT or errors in children or adults. However, 6–7 year-old children showed more commission errors in the C condition than in the SI condition. Children and adults showed response interference effects as reflected by slower RTs in the RI condition than the SI condition. In addition, children showed more commission errors in the RI condition than in the SI condition, whereas in adults commission errors were only marginally enhanced in the RI condition.

### Event-related potentials

In Figures 4 and 5, grand-average ERPs for the C and SI conditions are shown at electrodes where significant age effects of stimulus interference (SI minus C) were present or most pronounced, and whole scalp difference maps of the effects of stimulus interference in the different groups are shown in the time windows of interest. Similarly, Figures 6 and 7 show grand-average ERPs for the SI and RI conditions at electrodes where significant age effects of response interference (RI minus SI) were present or most pronounced, and whole scalp difference maps of the effects of response interference in the different groups are shown in the time windows of interest.

#### Stimulus interference effects on occipital P1 amplitude (80–140 ms)

As shown in Figure 4, a reduction of the P1 amplitude for the SI condition in comparison with the C condition over right-hemispheric and central occipital sites was most pronounced in 6–7 year-olds. This effect was confirmed by a Group × Condition interaction (F(3, 71) = 5.6, p = .002, epsilon = 1.0, *η*_*p*_^2 ^= .19). The non-significant three-way interaction with Electrode (Group × Condition × Electrode: F(6, 142) = 1.7, p = .15, epsilon = .65, *η*_*p*_^2 ^= .07) showed that this effect did not differ between the three electrodes and this factor was disregarded in further analyses. Separate tests for every group showed a significant reduction of the P1 amplitude in the SI condition in comparison with the C condition only in 6–7 year-olds (F(1, 17) = 11.7, p = .003, *η*_*p*_^2 ^= .41). There were no SI-C effects on the P1 amplitude in the other groups (8–9 year-olds: F(1, 18) < 1, p = .68, *η*_*p*_^2 ^= .01; 10–12 year-olds: F(1, 16) < 1, p = .33, *η*_*p*_^2 ^= .06; adults: F(1, 20) < 1, p = .35, *η*_*p*_^2 ^= .05).

#### Stimulus interference effects overlapping the negative (N4) and positive (P3) components (adults 260 – 400 ms; children 400 – 560 ms)

Stimulus interference effects were most pronounced in the ERPs of the youngest children and overlapped the negative N4 component and following positive component (P3). As shown in Figure 5 at representative electrode CPz, in 6–7 year-olds in a window from 400–560 ms the amplitude was more negative in the SI condition than in the C condition; this effects overlapped the negative and positive component, causing an enhanced negative component and a reduced positive component in the SI condition. As shown in the topographical difference maps, this negative amplitude difference of the SI condition minus the C condition was widely distributed over the scalp in 6–7 year-olds and appeared to decrease with age. This pattern was confirmed by ANOVA results showing a main effect of Condition (F(1, 71) = 11.7, p = .001, epsilon = 1.0, *η*_*p*_^2 ^= .14), and an interaction of Group × Condition (F(3, 71) = 3.5, p = .02, *η*_*p*_^2 ^= .13). As the interaction with Electrode was not significant (Group × Condition × Electrode: F(15, 355) < 1, p = .58, epsilon = .32, *η*_*p*_^2 ^= .03), this factor was disregarded in further analyses. Separate tests for every Group showed a Condition effect in 6–7 year-olds (F(1, 17) = 24.0, p < .0005, *η*_*p*_^2 ^= .59) across all electrodes (Condition × Electrode: F(5, 85) < 1, p = .54, *η*_*p*_^2 ^= .03). There were no condition effects in any of the other groups (8–9 year-olds: F(1, 18) = 3.8, p = .07, *η*_*p*_^2 ^= .17; 10–12 year-olds: F(1, 16) < 1, p = .52, *η*_*p*_^2 ^= .03; adults: F(1, 20) < 1, p = .65, *η*_*p*_^2 ^= .01).

#### Response interference: early effects on parietal-occipital positive component (440–540 ms)

As shown in Figure 6, the positive component over lateral parietal and parieto-occipital sites between 440–540 ms was reduced in amplitude for the RI condition in comparison with the SI condition in 10–12 year-olds and adults. This was confirmed by a Group × Condition × Electrode × Hemisphere interaction (F(3, 71) = 3.0, p = .04, epsilon = 1.0, *η*_*p*_^2 ^= .11). Separate tests for every Group showed a main effect of Condition in 10–12 year-olds (F(1, 16) = 5.6, p = .03, *η*_*p*_^2 ^= .26) and adults (F(1, 20) = 5.3, p = .03, *η*_*p*_^2 ^= .21), but not in the two younger groups (6–7 year-olds F(1, 17) < 1, p = .81, *η*_*p*_^2 ^= .003; 8–9 year-olds F(1, 18) < 1, p = .52, *η*_*p*_^2 ^= .02), and an interaction of Condition × Electrode × Hemisphere: F(1, 20) = 4.2, p = .05, epsilon = 1.0, *η*_*p*_^2 ^= .17) for adults. The latter indicated that the effect in adults was stronger over the left hemisphere than over the right hemisphere. Inspection of difference waves and within-group t-tests indicated that in 10–12 year-olds this amplitude reduction for the RI condition in comparison with the SI condition already started around 300 ms. Therefore, an additional window between 300–440 ms was entered in a mixed design ANOVA using the same factors and selection of channels as before. This analysis showed an interaction of Group × Condition (F(3, 71) = 3.1, p = .03, epsilon = 1.0, *η*_*p*_^2 ^= .12). Separate tests for every Group showed an amplitude reduction between 300–440 ms only in 10–12 year-olds (F(1, 16) = 7.5, p = .01, *η*_*p*_^2 ^= .32), but not in any of the other groups (6–7 year-olds: F(1, 17) < 1, p = .93, *η*_*p*_^2 ^= .001; 8–9 year-olds: F(1, 18) = 1.7, p = .21, *η*_*p*_^2 ^= .09; adults: F(1, 20) = 2.5, p = .13, *η*_*p*_^2 ^= .11).

#### Response interference: late effects on positive component across whole scalp (adults 680–800 ms; children 700–960 ms)

As shown in Figure 7, there was a second later effect of response interference on the descending flank of the positive component across the whole scalp; the amplitude was enhanced in the RI condition in comparison with the SI condition in all groups. This effect was confirmed by the ANOVA analyses showing a main effect of Condition (F(1, 71) = 13.4, p < .0005, *η*_*p*_^2 ^= .16), and no interaction of Group × Condition (F(3, 71) = 1.9, p = .13, *η*_*p*_^2 ^= .08) or Group × Condition × Electrode (F(15, 355) = 1.6, p = .15, epsilon = .37, *η*_*p*_^2 ^= .07).

Given the presence of the amplitude enhancement of the positive component in every group, and the discussion about the processes it reflects, additional correlation analyses were conducted between the size of the amplitude enhancement and behavioural measures of response interference. As there was no interaction with the factor Electrode, all electrodes that had been included in the ANOVA (Fz-F1-F2, FCz-FC1-FC2, Cz-C1-C2, CPz-CP1-CP2, Pz-P1-P2, Oz-O1-O2) were averaged to a "whole-scalp average". The whole-scalp average of the amplitude enhancement, computed as the whole-scalp average for the RI condition minus the whole-scalp average for the SI condition, was correlated, including all subjects, with the overt behavioral manifestations of response interference, computed as the difference in RT and errors for the RI condition and the SI condition. There was a significant positive correlation between the whole-scalp amplitude difference and the RT response interference effect (r(75) = 0.33, p = .004) indicating that RT response interference increased with amplitude difference; subjects with the highest late positive component amplitude increase in the RI (versus SI) condition showed the largest interference effects on RT. No correlation between this RI-amplitude increase and error increase for RI in comparison with SI was found (r(75) = 0.05, p = .68). Correlation analyses between the other ERP effects of stimulus interference and response interference and behavioural measures of stimulus and response interference were also conducted, but none of the other correlations were significant.

To summarize the ERP results, only the 6–7 year-olds showed ERP modulations related to stimulus interference. The P1 component over right-hemispheric and central occipital sites was reduced in amplitude for the SI condition in comparison to the C condition between 80–140 ms. In addition, the amplitude in the SI condition in comparison with the C condition showed a widely distributed enhancement of a negative component and a reduction of a positive component resulting in a negative amplitude difference for SI minus C between 400–560 ms in the youngest children. For response interference, an amplitude reduction was found for the RI condition in comparison with the SI condition of a positive P3-like component over lateral parietal and parieto-occipital sites between 300–540 ms in 10–12 year-olds and between 440–540 ms in adults. In adults this effect was stronger over the left hemisphere. In addition, there was a widely distributed amplitude enhancement of the late positive component between 700–960 ms in children and between 680–800 ms in adults in the RI relative to the SI condition. The size of this enhancement correlated positively with the size of the RT response interference effect.

## Discussion

The present study aimed to explore the development of stimulus interference control and response interference control in children aged 6–12 years and adults using a manual version of a color-object Stroop task. In the color-object Stroop task, line drawings of familiar objects were presented either in their canonical color or in another color (incongruent) and subjects classified the printed color of the stimuli by pressing one of two buttons. If objects are presented in another color than their canonical color, the irrelevant stimulus dimension "canonical color" of the object interferes with the relevant stimulus dimension "printed color" of the object. In the congruent (C) condition, objects were presented in their canonical color. In the stimulus incongruent (SI) condition, there was interference at the stimulus level but not at the response level as objects were presented in an incongruent color that was allocated to the same response button as the canonical color. In the response incongruent (RI) condition, there was interference at the stimulus level and at the response level as objects were presented in an incongruent color that was allocated to the response button opposite to the button associated with the canonical color. Stimulus interference was measured with a comparison of the SI condition and the C condition. Response interference was measured with a comparison of the RI condition and the SI condition. Children were allocated to one of three age groups (6–7, 8–9, 10–12 years old) to allow for a detailed examination of the developmental trajectory of interference control. ERPs were measured to examine the temporal course of these processes and characterize developmental changes in brain activation. Below, behavioral results and ERP results are related to each other for stimulus interference and response interference and the data are discussed in more detail.

### Development of stimulus interference control

There were no stimulus interference effects on RT or errors in children or adults. However, against expectations, 6–7 year-old children made more commission errors in the congruent condition than in the stimulus incongruent condition. This enhancement in commission errors was accompanied by an early P1 amplitude enhancement between 80–140 ms over right-hemispheric and central occipital sites in the congruent condition relative to the stimulus incongruent condition. No early P1 modulation was found for response interference (SI versus RI). Comparable ERP modulations around 100 ms with a right occipital maximum have been shown in other studies using object stimuli. In an object-decision task, atypical objects that violated conventional expectations evoked higher P1 amplitudes than typical objects [53]. Furthermore, recent visual repetition priming studies showed enhanced P1 amplitudes to targets preceded by unrelated as compared to related stimuli [54-56]. Such findings might indicate that when the visual features of a stimulus are more salient or less expected, they evoke a higher P1 response. In the present study, the P1 amplitude increase in the congruent condition in the youngest children may be due to the differences in the probability of occurrence of stimulus incongruent stimuli (SI and RI: 0.5 probability of occurrence) and congruent stimuli (0.25 probability of occurrence). These probability differences might unintentionally have caused congruent trials to be perceived as more salient or deviant in comparison with stimulus incongruent stimuli, evoking a higher P1 response in this condition, but only in 6–7 year-olds. Although speculatively, the presence of these effects only in 6–7 year-olds might be due to developmental differences in the strength of top-down processes that suppress such "novelty" responses, thereby preventing a preoccupation with the most salient events in older individuals. The lack of such higher-order control processes might also be responsible for the enhancement of commission errors to the less frequent congruent stimuli in 6–7 year-olds. Evidence for modulating effects of top-down cognitive control mechanisms on the P1 amplitude has been shown before (in adult subjects) [57].

In 6–7 year-old children the P1 amplitude reduction was followed by an amplitude enhancement of a negative (N4) component and an amplitude reduction of a positive (P3) component between 400–560 ms in response to stimulus incongruence of the printed color and the canonical color of the presented objects. This effect was widely distributed over fronto-central, centro-parietal, and parieto-occipital sites and was not present in older children or adults. Such a negative amplitude modulation for the incongruent condition around 400 ms has repeatedly been reported in Stroop ERP studies with healthy adult participants, and has been related to the process of conflict detection [31,32,34,35,38,39] and the need to suppress irrelevant conflicting information [36]. Although it mainly has been shown for combined stimulus-response interference, in one other Stroop ERP study this negative modulation was also elicited by interference at solely the stimulus level [38]. The absence of behavioral effects of stimulus interference in the present results suggests that stimulus interference control in 6–7 year-olds was already successful in solving conflict before its expression in behavior. The more negative amplitude in the incongruent condition may thus be a reflection not only of the detection of conflict but also of the implementation of control and conflict resolution. This has been suggested before in studies that used dipole fitting and showed that the more negative amplitude in the incongruent condition around 400 ms arose from activity in the anterior cingulate cortex (ACC) and the prefrontal cortex (PFC) [32,38]. Whereas the ACC is assumed to be related to conflict detection and evaluation, the PFC has been related to the implementation of control [58-61]. The activation of posterior (parieto-occipital and occipital) areas might be related to the detection of perceptual conflict in areas associated with object or color processing. Support for this comes from an fMRI study by Banich et al. [21] in which activation patterns evoked by color-incongruence in color-word Stroop and color-object Stroop tasks were compared. Whereas frontal activation was comparable in both tasks, in the object-Stroop there was enhanced activation in the ventral visual processing stream (areas associated with object processing) when the to-be-named color was incongruent with the canonical color of the presented object. In the color-word task enhanced activation was similarly found in areas associated with word processing. Also in other fMRI studies and ERP studies it has been shown that the areas activated and the scalp distribution of interference-related modulations, respectively, depend on the type of interference and the type of task stimuli [26,27,29,62]. The negative amplitude modulation for the incongruent condition in 6–7 year-olds may thus be a reflection of the detection of conflict and the implementation of control and conflict resolution. Finally, the broad scalp distribution of the interference-related negative amplitude difference in 6–7 year-olds is also in line with developmental fMRI studies showing that children recruit large and diffuse regions in tasks that require executive control while adults show more focal activation (for reviews, see [24,63]). The previous ERP Stroop studies that reported the negative amplitude difference were all conducted in adult samples, whereas the effect here was found in 6–7 year-olds.

### Development of response interference control

The behavioral results showed a similar RT delay in the RI as compared to the SI condition in children and adults, but children made more commission errors than adults in the RI as compared to the SI condition. These findings indicate that the task-irrelevant canonical color and the task-relevant printed color of the object activated conflicting response maps based on learned stimulus-response associations, and detection and resolution of this conflict resulted in a reaction time delay in all groups. The larger number of errors in children indicates that there were more instances in which they failed to inhibit the execution of the activated response map associated to the task-irrelevant canonical color of the object. It is important to note that the increase in errors in children cannot be explained by differences in processes of working memory (e.g. rule-holding) or response strategies since these would be expected to be comparable for stimulus incongruent and response incongruent conditions. Instead, children were worse in detecting response conflict, inhibiting incorrect response tendencies, selecting the correct response, or a combination of these. This is consistent with developmental studies showing immature response inhibition abilities in children between the ages of 4–13 years [43,64-67], and continuing developmental improvements in processes of cognitive control through adolescence [63]. Furthermore, a recent numerical Stroop study [42] similarly showed mainly interference due to response related processes as compared to stimulus processes in 9 and 11 year old children and argued that this was probably due to trouble with inhibition of response tendencies in children. All together these studies suggest that the developmental improvement in these processes of response inhibition occurs at a later age, during adolescence.

ERP results showed an amplitude reduction of a positive component around 400 ms over lateral parietal and parieto-occipital sites in the response incongruent condition relative to the stimulus incongruent condition, in 10–12 year-olds and adults. In adults this effect was stronger over the left hemisphere than over the right hemisphere. In other ERP Stroop studies, a similar reduction of a P3-like parietal component has repeatedly been shown, though mainly with a broader scalp distribution (e.g., [30,34,68]), and a similar left-hemispheric dominance for the effect in adult participants was shown by Lansbergen et al [30]. In these studies the amplitude reduction was related to the process of conflict detection [30,34,68] as well as the selection of competing responses [31]. However, in all these Stroop studies, combined stimulus-response interference was measured whereas the amplitude reduction in the present results was shown for solely response interference. In a recent numerical Stroop ERP study a similar reduced positive component over parietal sites was related to response interference as it occurred after the measured onset of motor preparation [40]. The parietal amplitude reduction might thus be related specifically to response interference. Although ERP results do not allow strong conclusions about sources based solely on scalp topography, some speculations can be made as parietal areas have been related to response conflict and response-related processes in a number of studies. Firstly, the parietal cortex has been suggested to contain the representation of task-relevant S-R associations and action codes [62,69]. An increase in activation in the left parietal cortex for incongruent trials in an fMRI flanker study with adult participants was suggested to be associated to the activation of competing response codes [62,69,70]. Secondly, left parietal activation has been related to attention to hand movements [71,72]. Using TMS, disturbance of the left parietal cortex mainly affected performance on trials that required subjects to disengage motor attention from the preparation of one movement to another [71]. Response incongruent trials in the present task were associated with a similar requirement. Taken together, these studies suggest that the parietal effect in 10–12 year-olds and adults is related to the activation of conflicting S-R associations or might reflect an increase in attention in response incongruent trials. Speculatively, this developmental parietal effect may be related to the developmental pattern of behavioural results showing a reduced ability of children to inhibit responses. That is, higher levels of attention and an improved ability to detect response interference might be responsible for the developmental between-group difference in response errors. As an alternative however it should be noted that overall response times and number of errors decreased linearly with age, indicating that general task performance increased across age. Therefore, the task may have required so much effort and attention allocation in 6–7 year-olds and 8–9 year-old children (as indicated by their slower RTs and increased error rates) that amplitudes were already at ceiling level in the SI condition. To be conclusive, further research is necessary.

The parietal amplitude reduction was followed by a late amplitude enhancement of a positive component for response incongruent trials as compared to stimulus incongruent trials that was widely distributed over the scalp in each of the groups. The assumed functional significance of the late positive component enhancement differs between studies, sometimes depending on the region where the effect was examined, and ranging from conflict detection and conflict resolution to response selection [34-36]. Source analyses studies have indicated that the positive component amplitude enhancement may arise from a distributed network involving lateral frontal, parietal, and occipital cortices [30,34]. In the present study the absence of the positive component amplitude enhancement for stimulus interference suggests a specific association with response interference. Indeed, correlation analyses indicated that the size of the amplitude enhancement (present across the whole scalp) was related to the size of the reaction time response interference effect. A specific relation of the positive component amplitude enhancement to response interference was also suggested by Szucs et al. [40] in a numerical Stroop task, as it occurred after the measured onset of motor preparation. Several other studies showed a relation of the amplitude enhancement to the size of conflict (larger amplitude enhancements in high conflict versions of the Stroop paradigm; [30,36]), or to the size of the reaction time interference effect [73], but in these studies stimulus and response interference were not separated. Rueda et al. [73] concluded that the (parietal) positive component amplitude enhancement might reflect the increase in evaluation of the incongruent stimuli that is necessary to determine the correct response.

Taken together the results for response interference showed a late positive component amplitude enhancement in every group that might be related to response conflict detection and resolution, in line with it's correlation with the response interference effect and the behavioural results that showed RT response interference in all the groups as well as resolution of conflict in the majority of trials. The earlier lateral parietal effect of response interference was only present in the oldest children and adults. This suggests a relatively late development starting around early adolescence. Interestingly, a similar developmental pattern of parietal activation was shown in an fMRI color-word Stroop study [74]. Stroop-related activation of the parietal and parieto-occipital cortex increased during childhood (7–11 years) and reached adult-like (18–22 years) levels in adolescence (12–16 years). The parietal modulation might be related to the reduced ability of children to control response interference that was shown here and in other studies [43,64-67].

It is important to note that whereas ERP measures can reveal the temporal course of interference with great sensitivity, the present data do not tell us how different activation patterns across the scalp are related; e.g. how involved brain networks and communication within such networks develop. In adults, parietal and prefrontal activity co-occur in the performance on a large number of cognitive tasks (for a review, see [75]), and fMRI studies have shown the importance of communication between prefrontal and parietal areas for adequate response inhibition and interference control (e.g., [21,69,75-79]). The development of networks in the brain proceeds slowly throughout late childhood and adolescence, consisting of structural changes like synaptic pruning, gray matter thinning, and myelination [80-84]. These developmental changes are thought to affect the efficiency of cognitive control [63]. For instance, in a recent developmental diffusion tensor imaging study [85] changes in frontostriatal connectivity over age (7–31 years) were paralleled by improvements in cognitive control in a go-nogo task. In two color-word Stroop studies that examined functional connectivity using EEG [86,87] higher and prolonged coherence within frontal and parietal areas was shown for the incongruent condition. This was interpreted as the recruitment and engagement of control to solve interference and select the correct response. The measures used in these studies seem of great additional value for future developmental Stroop studies as they reveal functional connectivity while preserving temporal precision. Such additional EEG measures might further explain differences in behavioral results as they reveal differences in communication.

## Conclusion

Using different types of Stroop tasks previous studies have shown that interference is stronger in children than in adults [11,13-15,17]. The results of the present study showed that this is also the case for the color-object Stroop task and therefore extend Prevor and Diamond's [16] results on a similar color-object Stroop task of interference in children between 3.5–6.5 years. More importantly, the current study mainly pointed in the direction of stronger response interference as opposed to stimulus interference in children than adults. Although in 6–7 year-old children interference at the perceptual stimulus-level elicited an early P1 reduction (80–140 ms) over occipital sites followed by a broadly distributed negative component amplitude enhancement and a positive component amplitude reduction (400–560 ms), there were no signs of stimulus interference in behavior. Stimulus interference control processes, possibly reflected by the broadly distributed negative amplitude difference, were already successful to prevent the expression of stimulus interference in overt behavior. Processes of stimulus interference control as measured with the color-object Stroop task thus seem to reach mature levels relatively early in childhood, around 6–7 years. Development of response interference control appears to continue into late adolescence as 10–12 year-olds were still more susceptible to errors of response interference than adults. The ERP results (parietal positive component amplitude reduction) suggest that this might be due to differences in the allocation of attention or an improved detection of response conflict in adults. A broadly distributed enhanced positive component that was present in every group most likely reflected processes of conflict detection and conflict resolution and appears to be specific to response interference.

## Appendix

^1 ^Two children from the 8–9 year-old group and two children from the 10–12 year-old group had scores on the Internalizing subscale that were in the borderline clinical range, and one child from the 6–7 year-old group and one child from the 8–9 year-old group had scores on the Attention subscale that were in the borderline clinical range.

^2 ^Familiar objects were objects with a canonical color red (heart, lips, lady-bird, and strawberry), green (tree, leaf, frog, and pear), yellow (sun, cheese, banana, and lemon), and grey (elephant, mouse, rhinoceros, and dolphin).

^3 ^Adults and children also performed on a flanker task. These data will be discussed somewhere else. Task order was balanced across participants.

^4 ^Analyses were reiterated on untransformed mean RTs, median RTs and individually standardized mean RTs ([88], p.788). All analyses showed a similar pattern of result (response interference but no stimulus interference).

## Authors' contributions

EMMJ performed EEG analysis and statistical analysis, interpreted the data and drafted and revised the manuscript. LMJ conceived of and supervised the study, contributed to the study design, data analysis, and interpretation, and helped with drafting and revising the manuscript. All authors read and approved the final manuscript.
